# Microfluidic Contact Lenses

**DOI:** 10.1002/smll.201704363

**Published:** 2018-03-09

**Authors:** Nan Jiang, Yunuen Montelongo, Haider Butt, Ali K. Yetisen

**Affiliations:** ^1^ School of Engineering and Applied Sciences Harvard University Cambridge MA 02138 USA; ^2^ Department of Chemistry Imperial College London South Kensington Campus London SW7 2AZ UK; ^3^ Universidad De La Salle Bajío León 37150 Mexico; ^4^ Nanotechnology Laboratory School of Engineering University of Birmingham Edgbaston Birmingham B15 2TT UK; ^5^ Institute for Measurement Systems and Sensor Technology Technische Universität München Theresienstrasse 90 80333 Munich Germany; ^6^ School of Chemical Engineering University of Birmingham Edgbaston Birmingham B15 2TT UK; ^7^ Institute of Translational Medicine Mindelsohn Way, Edgbaston Birmingham B15 2TH UK

**Keywords:** contact lenses, diagnostics, laser ablation, microfluidics, tear film

## Abstract

Contact lens is a ubiquitous technology used for vision correction and cosmetics. Sensing in contact lenses has emerged as a potential platform for minimally invasive point‐of‐care diagnostics. Here, a microlithography method is developed to fabricate microconcavities and microchannels in a hydrogel‐based contact lens via a combination of laser patterning and embedded templating. Optical microlithography parameters influencing the formation of microconcavities including ablation power (4.3 W) and beam speed (50 mm s^−1^) are optimized to control the microconcavity depth (100 µm) and diameter (1.5 mm). The fiber templating method allows the production of microchannels having a diameter range of 100–150 µm. Leak‐proof microchannel and microconcavity connections in contact lenses are validated through flow testing of artificial tear containing fluorescent microbeads (*Ø* = 1–2 µm). The microconcavities of contact lenses are functionalized with multiplexed fluorophores (2 µL) to demonstrate optical excitation and emission capability within the visible spectrum. The fabricated microfluidic contact lenses may have applications in ophthalmic monitoring of metabolic disorders at point‐of‐care settings and controlled drug release for therapeutics.

## 1 Introduction

Since the first soft contact lens was created in the 1970s, its composition has been continuously evolved to have permeability to oxygen and improve its hydrophilicity.^1^ Hydrogel‐based contact lens became a ubiquitous technology to correct vision and cosmetic purposes used by 150 million people worldwide.^2^ Contact lenses are worn over cornea of the eye, which allow continuously interacting with the eyeball and tear fluid. It is advantageous to fabricate the contact lens as a minimally invasive diagnostic device for continuously monitoring intraocular pressure and physiological state in real time.^3^, ^4^ Hence, the utilization of the contact lens as a potential minimally invasive platform for the quantitative analysis of tear is due to its readily availability, flexibility, and biocompatibility for in vivo diagnostics. Over the last decade, electrochemical sensors were integrated in contact lenses to measure the concentration of glucose for diabetes management.^5^ Another contact lens sensor, Triggerfish (Sensimed), has received food and drug administration approval for measuring intraocular pressure for the clinical management of glaucoma.^6^, ^7^ Bimetallic sensing‐resistive strain gauges were used as sensing materials to measure geometrical changes of corneoscleral junction correlated with intraocular pressure.^8^ The recorded signal was sent to wireless readout device that provided external power to the contact lens. Recently, an electronic contact lens with high transparency was fabricated to wirelessly measure the glucose concentration in tear fluid and intraocular pressure by electrochemical reactions simultaneously.^9^ However, most of the electronic contact lenses are limited due to the electrochemical signal drift as the eye blinks, have complicated fabrication, and subject to vision obstruction. While these studies demonstrated proof‐of‐concept sensing platforms, the correlation of blood glucose and tear glucose has not been clearly established.^10^ Other studies involved coating contact lenses with graphene to reduce electromagnetic field interference and induce dehydration protection.^11^

Replication methods, such as imprinting,^12^ injection‐molding,^13^ and polymer casting,^14^ have been developed to fabricate microfluidic devices utilizing templates with desired sizes and geometries. The microstructures of templates could transfer the patterns to a polymer substrate with high reproducibility.^15^ Commonly used SU‐8 photoresist and thermoplastic materials as the patterning templates are required, but they lack flexibility to produce curved surfaces.^15^ A rapid method to fabricate microstructures within the polymer substrate is to use flexible polymer threads that serve as templates to producing microchannels.^16^ However, the size variation and high surface roughness of threads limit their applications as templates in microchannel fabrication.

Conventional fabrication of microfluidic devices requires time‐consuming, laborious, and high‐cost soft photolithography.^17^ Direct laser ablation has been proposed as an alternative approach to fabricate microstructures on polymer surfaces.^18^ When the ablation intensity of a focused laser beam exceeds the level of binding energy of crosslinked polymer backbone, micropatterns can be formed on the polymer surface.^19^ CO_2_ laser ablation based on local explosive pyrolysis can enable the fabrication of micropatterns^20^ and precisely control the position of beam focal point on the polymer surface.^21^ As CO_2_ lasers operate in the mid‐infrared region (10.6 µm), water in the materials can absorb the beam energy that reduces risks of damage or the lens materials outside the focal point of the beam.^22^

Here, a facile and rapid fabrication is demonstrated to create a microfluidic contact lens through integrating of laser patterning and embedded templating. The fabricated microfluidic contact lenses comprised embedded microchannels connected to microconcavities. The depth and size of a laser patterned microconcavity in contact lenses were optimized by controlling laser ablation power and beam speed. The fabricated microconcavities in the contact lens were functionalized with fluorophores, where the fluorophore volume, crosslinking time, and stability were optimized. Each microchannel was formed by templating with a solid‐state optical fiber, which availed to artificial tear fluid flow. An optical fiber is a uniform template due to its flexibility, low surface roughness, and chemical inertness.^23^ The formed microchannels connected with the laser patterned microconcavities were embedded within the contact lens. The whole process of fabrication took ≈10 min. Fluorescent microbeads were utilized to study fluid flow through the microchannels to the microconcavities. The fabricated microfluidic contact lenses offer the potential to sample and detect biomarkers in the tear fluid for the diagnosis of eye diseases and for sequential and controlled drug release.

## 2 Results and Discussion

To understand and optimize the parameters that affect the formation of micropatterns in laser ablation, a time‐domain finite element method was used to simulate beam–polymer interaction. Laser ablation of a polymer matrix is a pure photothermal process where the matrix response to the beam can be explained as a result of elevated temperature.^24^ A beam spot size of 180 µm was adopted. Hydrogel was used as the ablation material that has thermal capacity of 1.4 × 10^3^ J kg^−1^ K^−1^ and density of 1.24 g mL^−1^. The temperature distribution within the hydrogel matrix is governed by the heat equation. Hence, the thermal energy *U* in the system is described in the numerical model as^25^

(1) $$Q=\frac{\partial U}{\partial t}-D\nabla^{2}U$$

where *Q* is the amount of heat source transferred from the laser and absorbed in the hydrogel matrix and *D* represents thermal diffusivity of the hydrogel matrix which equals to *k*/*ρC*_p_; where *ρC*_p_ is the volumetric heat capacity and *k* is the thermal conductivity of hydrogel matrix. The local heat *Q* can be extracted with the absorbed energy from the laser beam. According to the Beer–Lambert law, the radiant flux decreases as 10^−*zA*^ or *e* ^−*zα*^, where *z* is the beam penetration depth along the vertical direction, *A* is the absorbance, and *α = A ln* (*10*) and attenuation coefficient. If the laser beam is considered as a collimated 2D Gaussian function of total intensity *I_t_* (because the Rayleigh length which is significantly larger than the thickness of hydrogel matrix), the radiant flux per unit of area (*I*(*x*, *y*, *z*) can be expressed as

(2) $$I\left(x,y,z\right)=I_{t}N\left(x,y\right)e^{-z\alpha }$$

where the Gaussian envelop is

(3) $$N\;\left(x,y\right)=\frac{1}{2\pi \sigma }e^{-\frac{x^{2}+y^{2}}{2\sigma }}$$

and σ represents the characteristic standard deviation calculated from the spot size. The *I*(*x, y, z*) derivative with respect to the *z* axis provides the infinitesimal difference of energy which is the energy converted into heat. Hence, the heat energy is

(4) $$Q\left(x,y,z\right)=I_{t}N\left(x,y\right)z\alpha e^{-z\alpha }$$

**Figure 1:**
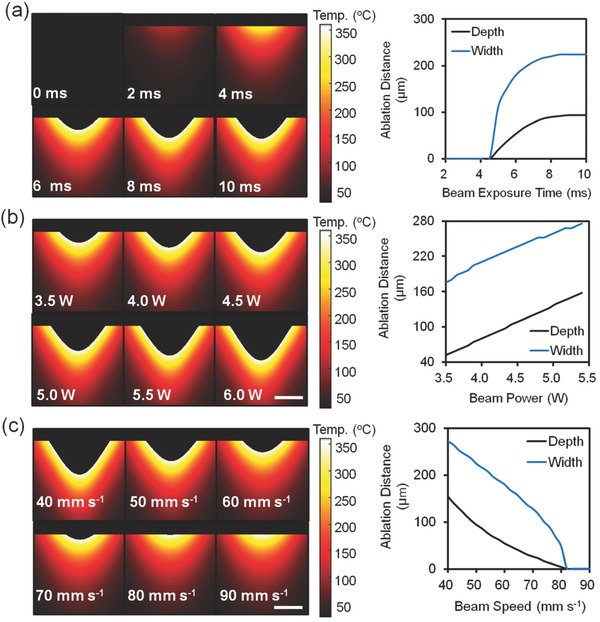
The simulated CO_2_ ablation of a hydrogel matrix. Temperature and 2D ablated concavity profiles variations with a) beam exposure time at a constant beam power of 4.3 W and beam speed of 50 mm s^−1^, b) power intensity at a constant beam speed of 50 mm s^−1^, and c) beam speed at a constant beam power of 4.3 W. Scale bars = 100 µm.

The temperature is extracted (*T = U·ρ·c_p_*) from Equation (4) and numerical solutions were computed through analysis in Matlab. **Figure** **1**a shows 2D computational model of temperature increase as a function of beam exposure time on hydrogel matrix (dynamic 3D model was shown in Video S1, Supporting Information). This process forms a microconcavity on the surface of the hydrogel matrix. As the exposure time increases from 2 to 8 ms, the simulated surface temperature increases to 360 °C and becomes stable with the prolonged exposure time (Figure 1a). As the exposure time prolonged from 0 to 10 ms, the ablation depth increased exponentially up to 94 µm, the ablation width increased up to 224 µm. The cooling effect from surroundings is negligible since the concavity forms within 8 ms. When the temperature reaches to decomposition temperature of hydrogel (360 °C),^26^ the matrix undergoes phase transition from solid state to gaseous state rather than temperature increase on the matrix surface. The phase transition results in mass loss, creating a concavity on the surface of the hydrogel matrix. The profile of the formed concavity originates from a nonlinear process. The radiant flux at the boundaries does not ablate the matrix, while it penetrates to deeper distances in the center. Figure 1b illustrates the temperature distribution and 2D profile of ablated concavity as a function of laser beam power. Increasing beam power from 3.5 to 5.4 W increased the ablation depth and width threefold and 1.6‐fold, respectively. When the beam exposure speed increased from 40 to 90 mm s^−1^, the ablation depth and width of concavity decreased from 140 and 264 µm to none (Figure 1c).

**Figure 2:**
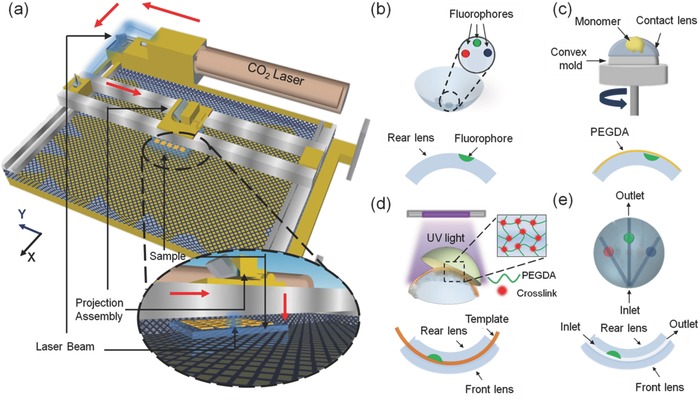
Fabrication and cross‐sections of microfluidic contact lenses. a) Schematic representation of CO_2_ laser patterning. Arrows show the laser path. b) Depositing fluorophores within the patterned microconcavities in the contact lens. c) Spin coating the contact lens with a PEGDA monomer layer. d) Placing fiber templates to form microchannels across the microconcavities and combining the fiber templates and microconcavities on the contact lens with a pristine contact lens by UV‐initiated free‐radical polymerization. e) Extracting the fiber templates from the contact lens to obtain a microfluidic contact lens.

CO_2_ laser ablation (10.64 µm, 30 W) with a spot size of ≈180 µm was chosen to pattern microconcavities in contact lenses due to its rapid fabrication and programmable beam accuracy and speed (**Figure** **2**a). Two pieces of commercial contact lenses were used for the microfluidic contact lens fabrication. In the first step, microconcavities were patterned on a contact lens by the laser ablation, followed by the deposition of fluorophores within microconcavities (Figure 2b). Subsequently, a poly(ethylene glycol) diacrylate (PEGDA) monomer layer was spin‐coated on the contact lens fixed on a convex mold (Figure 2c).

Silica fiber templates were placed within the PEGDA layer above the patterned microconcavities, sandwiched by another pristine contact lens as the front surface. The front and rear contact lenses were bound via UV‐initiated free‐radical polymerization (Figure 2d). Finally, the fiber templates were extracted from the contact lens to form the microfluidic device (Figure 2e).

**Figure 3:**
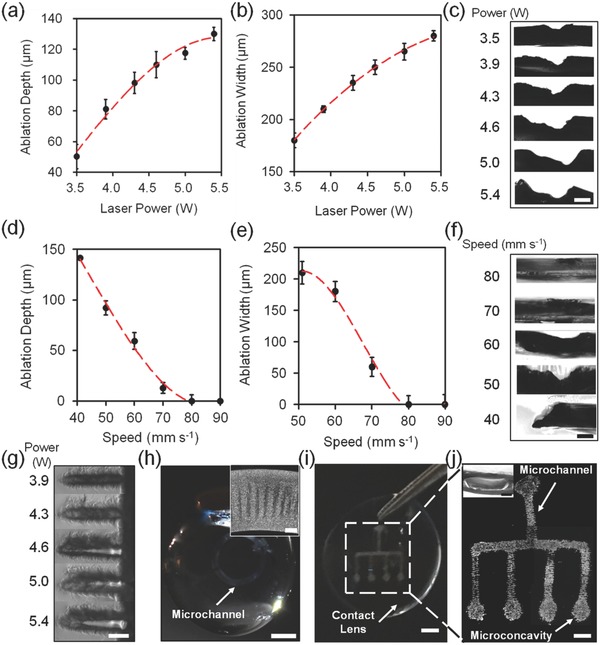
Laser‐ablated micropatterns in contact lenses. a) Depth and b) width of the laser‐ablated regions as the laser beam power increased from 3.5 to 5.4 W at a constant beam speed of 50 mm s^−1^. c) Cross‐sections of laser patterned microconcavity in contact lenses as the laser beam power increased at a constant beam speed of 50 mm s^−1^. Scale bar = 100 µm. d) Depth and e) width of the ablated microconcavity regions as the laser beam speed increased from 40 to 90 mm s^−1^ at a constant laser beam power of 4.3 W. f) Cross‐sections of laser patterned microconcavities in contact lens as the beam speed increased at a constant beam power of 4.3 W. Scale bar = 100 µm. g) Top view of laser patterned microconcavities as the laser beam power increased from 3.9 to 5.4 W. Scale bar = 300 µm. h) Photographs of a laser patterned circular microchannel in the contact lens matrix. Scale bar = 300 mm. Inset shows the magnified patterned microchannel. Scale bar = 160 µm. i) Photograph of a microfluidic contact lens array by the laser patterning. Scale bar = 200 mm. j) Microscopic image of laser patterned microfluidic array in the contact lens. Scale bar = 1 mm. Inset shows the cross‐section microscopic image of the patterned microchannel sealed with another pristine contact lens. Scale bar = 100 µm. Error bars represent the standard error (*n* = 3).

To produce microconcavities in the contact lens, a CO_2_ laser cutter having programmable laser power and beam speed were used. At power threshold values lower than 3.5 W with the constant speed at 50 mm s^−1^, the laser beam did not ablate the contact lens. As the power of the CO_2_ laser beam was increased from 3.5 to 5.4 W with a constant beam speed at 50 mm s^−1^, the microconcavity ablation depth increased from 48 to 130 µm (**Figure** **3**a), and the width of ablated patterns increased from 180 to 280 µm (Figure 3b). The ablated microconcavity depth depended on laser beam wavelength, exposure duration, and absorption by the contact lenses. Figure 3c illustrates the cross‐section microscopic images of the laser patterned microconcavities in contact lenses with a constant beam speed at 50 mm s^−1^. As the CO_2_ laser beam speed was increased from 40 to 90 mm s^−1^, the ablated concavity depth decreased from ≈140 µm to through cut (Figure 3d) and the patterned concavity width decreased from 210 µm to through cut at a constant power at 4.3 W (Figure 3e). Figure 3f illustrates the cross‐section photographs of the concavity in contact lenses patterned at beam speeds from 40 to 80 mm s^−1^ at a constant beam power (4.3 W). The through ablation cutting of laser power was 6 W with an ablation speed at 50 mm s^−1^, respectively. Figure 3g shows the optical microscopic images of the top view of laser patterned microconcavity in the contact lenses as the laser power increased from 3.9 to 5.4 W. These results were consistent with simulations that the increase of laser beam power from 3.5 to 5.4 W and the decrease of beam speed from 90 to 40 mm s^−1^ yield the increased ablation depth and width of concavity (Figure 1b,c).

Utilizing the optimized laser ablation engraving at a laser power at 4.3 W and beam speed at 50 mm s^−1^, a circular microchannel with a ≈800 µm channel width was patterned in the contact lens (Figure 3h). The inset in Figure 3h shows a magnified microscopic image of the fabricated microchannel. Figure 3i,j shows a patterned microfluidic array with one main microchannel having channel depth and width at 100 and 600 µm channeling into four other microchannels. The microchannel (depth = 100 µm) was produced at each channel outlet as the potential separated region.

**Figure 4:**
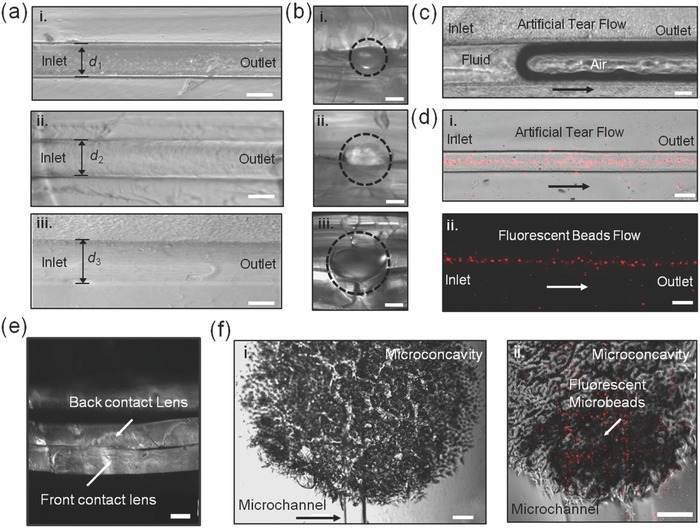
Characterization of fluid flow (100 µL h^−1^) in microfluidic channels embedded within contact lenses. a) Top‐view optical microscopic images of microchannels with different channel widths. Scale bar = 100 µm. b) Cross‐section microscopic images of the microchannels. *d*_1_ < *d*_2_ < *d*_3_. Dash circles show the microchannels. Scale bar = 50 µm. c) Artificial tear fluid flow within the microchannel. Scale bar = 50 µm. d) Microscopic images of artificial tear containing fluorescent beads within the microchannel. Scale bar = 100 µm. e) Microscopic images of sealed contact lenses. Scale bar = 100 µm. f) (i) Microscopic image of a microconcavity connected with a microchannel. Red fluorescence in (ii) shows artificial tear fluid flowing from the microchannel to the microconcavity. Scale bar in (i) = 100 µm. Scale bar in (ii) = 50 µm.

A silica fiber was used as a template to form a microfluidic channel in the contact lenses. Three fibers were placed over the convex side of a contact lens which was previously spin‐coated with PEGDA (100 vol%) and 2‐hydroxy‐2‐methylpropiophenone (2‐HMP) monomers as a thin layer (20 µm). A pristine contact lens was placed over the patterned contact lens to sandwich the fibers for templating. After UV‐initiated free radical polymerization, the fibers were extracted from the contact lens, yielding a microchannel within the contact lens (**Figure** **4**a). The fabricated microchannel width was controlled by templating with fibers having different diameters from 100 to 150 µm (Figure 4b). The microconcavities were sandwiched between the two contact lenses sealed with a PEGDA hydrogel layer. The fiber templated microchannel had lower roughness than the laser‐patterned microchannel (Figure 3h). Figure 4c shows the flow (100 µL h^−1^) of artificial tear fluid from an inlet toward an outlet within a single microchannel. Fluidic dynamics of the microfluidic channels in contact lenses were demonstrated by infusing fluorescent microbeads (1:9, v/v) in artificial tear fluid using a syringe pump. The captured fluorescence microscopic images show laminar flow within the microchannel (channel width = ≈100 µm) in the contact lens (Figure 4d). The fluid flowing without significant leakage in contact lens was due to the intact sealing of two contact lenses with an approximate thickness of 280 µm (Figure 4e). Figure 3f demonstrates a microconcavity (*Ø* = 1.5 mm) connected to a microchannel, in which artificial tear fluid containing red fluorescent microbeads (*Ø* = 1–2 µm) were flowing (100 µL h^−1^) into to the microconcavity. Microfluidic contact lens requires high transparency for clear eyesight. Transparent PEGDA serving as the layer for bonding two pieces of contact lenses satisfied this requirement (Figure S1a, Supporting Information). Moreover, when the microchannels (100–150 µm) were filled with tear fluid, the low refractive index contrast (<0.10) between contact lens (≈1.42)^27^ and tear fluid (≈1.34)^28^ would not significantly affect light transmission through the lens (Figure S1b, Supporting Information). The engraved microconcavities were slightly visible and distributed eccentrically, out of the line of sight (Figure S1b,c, Supporting Information). Furthermore, oxygen permeability is also an important physical parameter of the contact lens as it affects oxygen transfer from the ambient air to the ocular surface. Insufficient oxygen permeability of the contact lens may cause corneal edema.^29^ The PEGDA layer had lower oxygen permeability (2.2–2.9 barrer at a thickness of 1 mm) as compared to commercial contact lens materials (≈14 barrer at a thickness of ≈140 µm);^30^ however, PEGDA had high water content^31^ and the PEDGA layer was only ≈20 µm.

Additionally, the increased thickness of the microfluidic contact lens (from ≈140 to ≈280 µm) may also affect the wearing comfort factors such as decreased oxygen permeability. However, the fabricated contact lens was sufficient for wearability as compared to a commercial soft contact lens (≈400 µm).^32^ Therefore, the fabricated microfluidic contact lens would have sufficient oxygen permeability for ocular applications.

**Figure 5:**
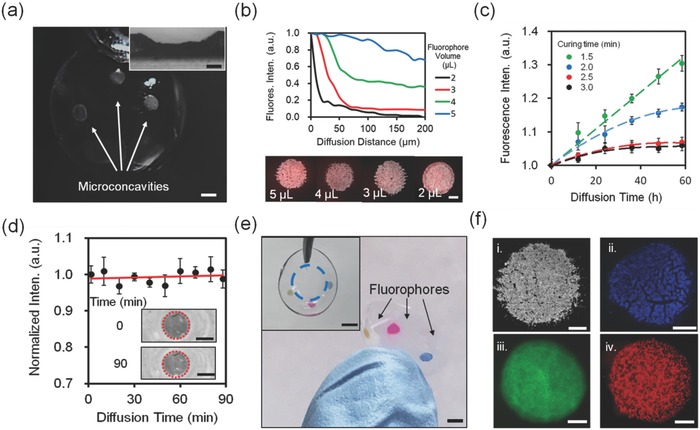
Characterization of fluorophore‐functionalized microconcavities in the contact lens. a) A photograph of laser‐patterned microconcavities. Scale bar = 1.5 mm. Inset shows the cross‐section of a microconcavity. Scale bar = 100 µm. b) Diffusion of rhodamine B away from the microconcavities over 3 min at 24 °C. The bottom microscopic images show the top views of fluorophore‐functionalized microconcavities. c) Fluorescence intensity of fluorophore as the diffusion time lapsed from 0 to 60 min, where the fluorophore polymerization time was increased from 1.5 to 3.0 min. d) Stability of immobilized fluorophore within the microconcavity as the time prolonged to 90 min. The inset shows the monochromatic photographs of the fluorophore mixture diffusion at 0 and 90 min. Red dashed circles show microconcavity boundaries. e) Fluorescent probes (diaza‐18‐crown‐6, fluorescein, and rhodamine B deposited in the microconcavities on the lens. The inset shows the top view. Scale bar = 2.0 mm. Blue dashed circle shows the transparent area for eye vision. f) Microscopic images of fluorescent probes (0.2 µL, 50 µmol L−1) emissions from the microconcavities (*Ø* = 1.5 mm, depth = 100 µm): (i) an empty microconcavity. (ii) fluorescent diaza‐18‐crown‐6; (iii) fluorescein; and (iv) rhodamine B. Scale bar = 500 µm.

Human tear fluid can be used as a surrogate for blood fluid by due to plasma leakage for application in real‐time analyte monitoring.^3^ To achieve multiple biomarker sensing or release multiple drugs for therapeutics, the fluorophores/drug molecules are of importance to be immobilized within the microconcavities without leakage and crosstalk in the contact lens. To demonstrate this capability, the contact lens microconcavities having diameters of 1.5 mm and depths of 100 µm were immobilized with fluorophores (**Figure** **5**a). A monomer solution containing 2‐hydroxyethyl methacrylate (HEMA) (98.0 mol%) and ethylene glycol dimethacrylate (EDMA) (2.0 mol%) was prepared to serve as a porous matrix for the fluorophore immobilization. This monomer solution was mixed (1%, v/v) with 2‐HMP in distilled water. The resulting monomer solution was mixed (1:2, v/v) with rhodamine B solution (50 µmol L^−1^). The fluorescent monomer mixture was deposited to the patterned microconcavity in contact lenses and crosslinked by UV polymerization. The fluorescent mixtures were added to the microconcavities in the contact lens and the volume was optimized to avoid leakage and interference. The diffusion of rhodamine B into the contact lens was measured as an optical probe model using a fluorescence microscopy. Figure 5b shows the diffusion plots of rhodamine B spreading beyond the microconcavity. Figure 5b inset shows the top‐view microscopic images of microconcavities with diffusing outward gradient of rhodamine B extending away from the channel boundary. As the contact lens matrix was homogeneous, the fluorescent molecular motion was not directional. Nevertheless, there was a net transfer from high concentrated to low concentrated region. As the aqueous mixture increased from 2 to 5 µL, the fluorescence intensity of rhodamine B increased five fold at the diffusion distance of 50 µm. The fluorescent mixture with a volume of 2 µL showed lowest fluorescence intensity which was decreased to zero at a diffusion distance of 150 µm over 3 min. 2 µL was found to be the optimized fluorophore volume, which could prevent the crosstalk among microconcavities.

The duration of fluorophore mixture polymerization was optimized to prevent spreading of the fluorophore through the microconcavity in the contact lens. A monomer mixture containing rhodamine B (50 µmol L^−1^, 0.2 µL) and HEMA, EDMA, and 2‐HMP was deposited in the microconcavities and exposed to UV light for 1.5–3.0 min. The contact lens was immersed in phosphate buffer solution (PBS) (10 mL, pH = 7.4). The effect of polymerization duration was evaluated by measuring the fluorescence intensity of released mixture in the PBS solution (Figure 5c). When the mixture was polymerized for 1.5 and 2.0 min, the fluorescence intensity of released mixture increased 1.29‐fold and 1.16‐fold respectively with diffusion time over 60 h. The increased fluorescence intensity indicated the continuous release of uncured rhodamine B‐contained mixture from the polyHEMA (pHEMA) hydrogel network. As the fluorophore monomer polymerization time increased to 2.5 and 3.0 min, the fluorescence intensity reached saturation after 24 h, which demonstrated that the fluorophore mixture was fully crosslinked within the microconcavities of the contact lens. The 5% increase in the fluorescence intensity in the first 12 h was due to the excess diffusion of rhodamine B molecules from the mixture. Since long‐term UV exposure time resulted in photobleaching, the optimal polymerization time for fluorophore mixture in the microconcavity was 2.5 min.

The stability of crosslinked fluorophore was studied by time‐lapse diffusion of rhodamine B within pHEMA hydrogel in the microconcavity (Figure 5d). Photographs (black and white) of rhodamine B in microconcavities were converted to gray images (Figure 5d inset). Gray values at the periphery of microconcavities were correlated to fluorescence intensity of diffused rhodamine B in the contact lens matrix. As the diffusion time prolonged to 90 min, the fluorescence intensity was found to be stable within the microconcavities. Three different fluorophores (50 µmol L^−1^, 0.2 µL) that are fluorescent diaza‐18‐crown‐6 (λ_ex_/λ_em_: 365/500 nm), fluorescein (λ_ex_/λ_em_: 492/525 nm), rhodamine B: λ_ex_/λ_em_: 553/627 nm) were deposited into microconcavities (*Ø* = 1.5 mm, depth = 100 µm). The original morphology of the contact lens was maintained with three small sensing regions near the edges which would not obscure vision (Figure 5e). Fluorescent probes were well dispersed in the microconcavities (Figure 5f). No diffusion or leakage was observed, demonstrating that the different fluorescent probes can be maintained in the microconcavities on the contact lens matrix without crosstalk.

## 3 Conclusion

To meet the requirements of future medical applications, the microfluidic contact lens could be improved from fabrication techniques. The front and rear contact lenses were sealed by weak interactions, which may cause leakage in long‐term use. The fabrication of microfluidic contact lens could be improved by using a femtosecond laser ablation used in cataract and LASIK surgeries and^33^ to reduce heat dissipation and subsequently créate accurate micropatterns. The microconcavities or microchannels could be precisely and rapidly created by focused laser ablation within a single contact lens, eliminating contact lens matrix damage and increased lens thickness and weight, and could therefore be used to fabricate microfluidics. However, this femtosecond laser‐based method requires high‐cost laser equipment and the deposition of fluorophores or drugs may become challenging.

Over the last 2 decades, the capabilities of microfluidics have been expanded to sample pumps,^34^ valves,^35^ storage,^36^ and mixers.^37^ Unlike discrete sampling at specific time points, microfluidic enables continuous fluid analyses.^38^ The integration of microfluidic capabilities in contact lenses is highly desirable to achieve precise tear fluid collection, sample preparation, and analyses in vivo or in vitro. Microfluidic contact lenses can enable continuous and controllable fluid collection, and storage for multiple analyses.^39^ The tear fluid could be dragged into microfluidic channels by capillary force. However, when the volume of tear fluid is not sufficient for diffusion, such as dry eye case, an autonomous pump, such as branched microstructure^40^ or liquid encapsulation design^34^ could be implemented within microfluidic contact lens by tuning the pressure difference within the microchannel. The immediate diffusion of tear fluid may produce erroneous sampling due to the saline solution in situ and reflexive tearing. A thermosensitive and dissolvable polymer could be embedded at the inlet of the microfluidic lens serving as a valve to delay the sample collection in vivo.^35^ By incorporating a sample collection region with preloaded desiccant within the microchannels in the contact lens, the microfluidic contact lens would allow the storage of collected tear fluid for a long term.^36^ The preserved tear fluid after recovery can provide more metabolic information by follow‐up tests such as sequencing and viral load measurements. Moreover, to enhance the flowing biomarkers in the tear fluid mixing with fluorophores under dynamic nonequilibrium conditions, turbulence and nonturbulence mixers could be incorporated in the microfluidic contact lens design to improve the accuracy of real‐time measurement.^29^, ^35^ With the development of emerging wearable technology, the microfluidic contact lens with stretchability and flexibility could also be fabricated by thermoplastic polymer.^7^

To expand the applicability of the microfluidic contact lens, colorimetric and fluorescent optical sensors could be crosslinked to the microconcavities for multiplex sensing capabilities.^41^ Chelating agent monomers can be immobilized as pendant groups in contact lenses for creating highly selective biosensors.^42^ The colorimetry and fluorescence intensity of the sensing regions could report on biomarker concentrations by using an encoded smartphone camera processing that converts complementary metal‐oxide‐semiconductor data to biomarker concentration values. The demonstrated method using a combination of laser patterning and fiber templating allowed rapidly and precisely fabricating microfluidic contact lenses. Contact lens sensors integrated with smartphone technologies may open the possibilities for real‐time proteomic and genomic analyses combined with treatment capabilities such as drug delivery.^43^ This technology can be extended to create a localized ion map under the microfluidic lens, continuously monitor allergens, and test systemic diseases including kidney function. With over hundred unanalyzed biomarkers in tear fluid,^44^ the exploitation of quantitative analysis through the microfluidic contact lens technology has the potential to create new diagnosis and treatment options in personalized medicine.^45^

## 4 Experimental Section

*Materials*: All chemicals were of analytical grade and used without further purification. 4‐[6‐[16‐[2‐(2,4‐dicarboxyphenyl)‐5‐methoxy‐1‐benzofuran‐6‐yl]‐1,4,10,13‐tetraoxa‐7,16‐diazacyclooctadec‐7‐yl]‐5‐methoxy‐1‐benzo (fluorescent diaza‐18‐crown‐6, high performance liquid chromatography purity ≥ 90%), λ_ex_/λ_em_: (346/500 nm); rhodamine B ([9‐(2‐carboxyphenyl)‐6‐diethylamino‐3‐ xanthenylidene]‐diethylammonium chloride) λ_ex_/λ_em_: (553/627 nm); fluorescein (λ_ex_/λ_em_: (490/525 nm). Contact lenses (1.0°, 6.0°, diameter: 1.4 cm, 1 d, Acuvue Moist) were purchased from Johnson & Johnson. PEGDA (*M*_w_ = 700 Da), EDMA (98%), HEMA (98%), and 2‐HMP (97%) were purchased from Sigma‐Aldrich. Red fluorescent microbead solution was purchased Createx Colors (East Granby, CT).

*Equipment*: A CO_2_ laser (VLS 2.30) operating at a wavelength of 10.64 µm at 30 W was purchased from Universal Laser Systems. An optical microscope (AXIO Observer D1) with phase contrast and fluorescence imaging was purchased from Zeiss. UV lamp (OmniCure S2000, λ = 350 nm, power: 850 mW) was purchased from Excelitas Technologies. A spin coater (WS‐650Mz‐23) was purchased from Laurell Technologies Corporation. Convex poly(methyl methacrylate) (PMMA) contact lens molds were custom designed and used as spin coating and photopolymerization supports. A programmable syringe pump (NE4000, New Era Pump Systems) was used for artificial fluid flow. SolidWorks (×64), CorelDRAW (×7), and Image J were used for design and image processing.

*Laser Patterning of Microconcavities in Contact Lenses*: Microconcavities were fabricated on the contact lens using a laser cutter. A CO_2_ laser cutter operating at a wavelength of 10.64 µm at 30 W was used to pattern contact lenses. Images were designed in CorelDRAW (insert version) as lines and surfaces. The power of laser beam was varied from 3.5 to 5.4 W at beam speeds ranging from 40 to 90 mm s^−1^.

*Channel Templating and Bonding of Contact Lenses*: The engraved rear contact lens with three microconcavities was placed on a convex PMMA mold. Silica fibers with a diameter of 100 µm were fixed across the engraved microconcavities and clamped on the PMMA support. A monomer solution was prepared by mixing (2:1, v/v) PEGDA and 2‐HMP (1 vol%) in deionized water. Mixture containing (5 µL) was pipetted on the concave side of a contact lens (thickness: 140 µm) surface, which was placed on a concave surface. The monomer solution was spin coated (4000 rpm, 45 s) on the contact lens to form a thin layer. The convex side of another contact lens was placed on the thin layer (thickness = 20 µm) of monomer solution and the system was UV‐crosslinked for 2.5 min. Finally, the templates were extracted from the contact lens.

*Characterization of Contact Lenses*: The diffusion of rhodamine B (500 µmol L^−1^) into the contact lens and bonding polymer was measured to quantify the area of dispersion to avoid cross talk between the solutions in the microconcavities. Fluid flow characterization in microchannels of contact lenses was also carried out. Artificial tear fluid containing microbeads (1 wt%) was injected into fabricated microfluidic contact lens using a single syringe pump (NE‐1010) at a flow rate of 100 µL h^−1^. Fluid flow within the contact lens was imaged by a fluorescence microscope at an excitation wavelength of 555 nm.

## Conflict of Interest

The authors declare no conflict of interest.

## Supporting information

Supplementary

Supplementary
