# Supplementary material for: Microfluidic Contact Lenses
Source: Small. 2018 Mar 9;14(15):1704363. doi: 10.1002/smll.201704363 (PMC6607692; doi:10.1002/smll.201704363)
Supplement: Supplementary file 1 — Supplementary [file SMLL-14-na-s002.pdf]

Copyright WILEY-VCH Verlag GmbH & Co. KGaA, 69469 Weinheim, Germany, 2018.

NANO small MICRO

Supporting Information

for *Small*, DOI: 10.1002/smll.201704363

Microfluidic Contact Lenses

*Nan Jiang, Yunuen Montelongo, Haider Butt, and Ali K. Yetisen**

SUPPORTING INFORMATION

Microfluidic Contact Lenses

Nan Jiang,[1] *Yunuen Montelongo,*[2] *Haider Butt,*[3] *Ali K. Yetisen*[4,5,6,]*

[1] School of Engineering and Applied Sciences, Harvard University, Cambridge, Massachusetts 02138, USA

[2] Department of Chemistry, Imperial College London, South Kensington Campus, London SW7 2AZ, UK

[3] Nanotechnology Laboratory, School of Engineering, University of Birmingham, Birmingham B15 2TT, UK

[4] School of Chemical Engineering, the University of Birmingham, Edgbaston, Birmingham B15 2TT, UK

[5] Institute of Translational Medicine, Mindelsohn Way, Edgbaston, Birmingham, B15 2TH, UK

[6] Harvard-MIT Division of Health Sciences and Technology, Harvard University and Massachusetts Institute of Technology, Cambridge, Massachusetts 02139, USA

KEYWORDS: Microfluidics; contact lenses; laser ablation; tear film; diagnostics

e-mail: akyetisen@gmail.com

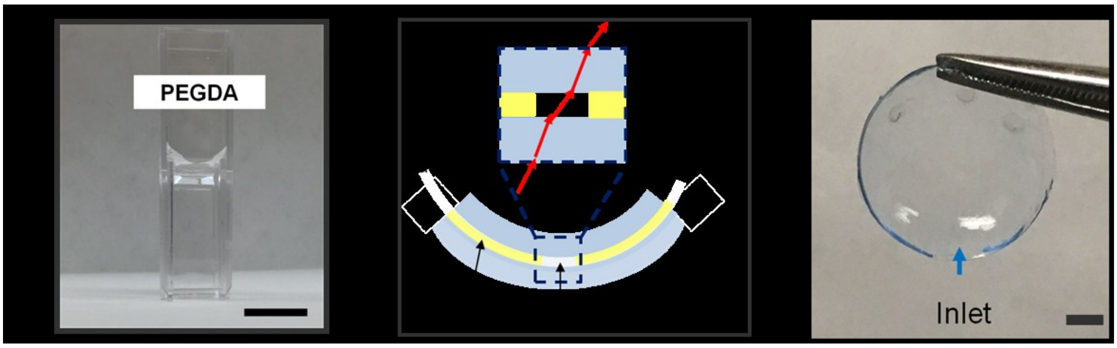

Figure S1. Evaluation of transparency of the fabricated microfluidic contact lens. (a) A photograph of the polymerized PEGDA hydrogel. Scale bar=1 cm. (b) Schematic of microfluidic contact lens cross-section. Red arrows show light propagation through the microfluidic contact lens. (c) Top view of microfluidic contact lens. Scale bar= 3 mm.
